# Microbiome analysis and biocontrol bacteria isolation from rhizosphere soils associated with different sugarcane root rot severity

**DOI:** 10.3389/fbioe.2022.1062351

**Published:** 2022-12-16

**Authors:** Xinyang Li, Yue Liu, Ziting Wang, Chenglong Yang, Runzhi Zhang, Yibao Luo, Yuming Ma, Yizhen Deng

**Affiliations:** ^1^ State Key Laboratory for Conservation and Utilization of Subtropical Agro-Bioresource, Guangxi Key Laboratory of Sugarcane Biology, Guangxi University, Nanning, China; ^2^ Laboratory of Crop Physiology and Field Ecology, Northwest A&F University, Yangling, China; ^3^ State Key Laboratory for Conservation and Utilization of Subtropical Agro-Bioresources, Guangdong Province Key Laboratory of Microbial Signals and Disease Control, Integrative Microbiology Research Centre, South China Agricultural University, Guangzhou, China

**Keywords:** sugarcane root rot, rhizosphere soil microecology, biocontrol bacteria, growth-promoting bacteria, fusarium

## Abstract

To explore the causal pathogen and the correlated rhizosphere soil microecology of sugarcane root rot, we sampled the sugarcane root materials displaying different disease severity, and the corresponding rhizosphere soil, for systematic root phenotype and microbial population analyses. We found that with increased level of disease severity reflected by above-ground parts of sugarcane, the total root length, total root surface area and total volume were significantly reduced, accompanied with changes in the microbial population diversity and structure in rhizosphere soil. Fungal community richness was significantly lower in the rhizosphere soil samples from mildly diseased plant than that from either healthy plant, or severely diseased plant. Particularly, we noticed that a peculiar decrease of potential pathogenic fungi in rhizosphere soil, including genera *Fusarium*, *Talaromyces* and *Neocosmospora*, with increased level of disease severity. As for bacterial community, Firmicutes was found to be of the highest level, while Acidobacteria and Chloroflexi of the lowest level, in rhizosphere soil from healthy plant compared to that from diseased plant of different severity. FUNGuild prediction showed that the proportion of saprophytic fungi was higher in the rhizosphere soil of healthy plants, while the proportion of pathogenic fungi was higher in the rhizosphere soil of diseased plants. By co-occurrence network analysis we demonstrated the *Bacillus* and *Burkholderia* were in a strong interaction with *Fusarium* pathogen(s). Consistently, the biocontrol and/or growth-promoting bacteria isolated from the rhizosphere soil were mostly (6 out of 7) belonging to *Bacillus* and *Burkholderia* species. By confrontation culture and pot experiments, we verified the biocontrol and/or growth-promoting property of the isolated bacterial strains. Overall, we demonstrated a clear correlation between sugarcane root rot severity and rhizosphere soil microbiome composition and function, and identified several promising biocontrol bacteria strains with strong disease suppression effect and growth-promoting properties.

## Introduction

Sugarcane (*Saccharum officinarum* L. cv. Badila) is an important economic crop as well as a popular tropical/subtropical fruit grown in tropical and subtropical regions, including China (Boaretto et al., 2021; Ren et al., 2022) (Supplementary Figure S1A). In recent years, with ratoon planting of sugarcane, a large number of pathogens that cause sugarcane root rot accumulate in plants or in the soil year by year, resulting in the imbalance of soil microbial community, which seriously restricts the development of the sugarcane industry (Pang et al., 2021; Ren et al., 2021). Among them, sugarcane root rot caused by *Fusarium commune* (Wang et al., 2018) occurs seriously in the chewing cane producing areas in Guangdong Province, China (Supplementary Figure S1A). Mild disease symptoms include wilt and yellow leaves, slow growth and reduction in tiller numbers of seedlings. In severe cases, the disease causes sugarcane death and large-scale yield reduction, which poses a serious threat to the high yield and high sugar content in sugarcane (Ren et al., 2021).

The root system is the main organ of crops to anchor and support the above-ground parts of plants and to absorb soil water and nutrients, and is closely related to the growth and development, physiological functions and material metabolism of plants. If the root system of plants is under adverse conditions, a series of morphological changes will occur (Potocka and Szymanowska-Pulka., 2018; Vives-Peris et al., 2020). On the other hand, rhizosphere soil microbiota, as the most active organisms in the soil, form a dynamic combination of plants and the interactive environment between soil-rhizosphere microbiota-plants (Peiffer et al., 2013). If the balance between soil-rhizosphere microbiota-plants is disrupted, plant growth would be affected (Qu et al., 2022). Microbial pathogens are sharing the same rhizospheric environment with other microbes, therefore encountering the same abiotic environmental factors (Mendes et al., 2013). Correspondingly, physiological changes in the microbes may also affect each other, thus resulting in a dynamic micro-ecological environment around the root system. Disease occurrence could be viewed as an outcome of such micro-ecological interaction (Xu et al., 2012). The rhizosphere microbiota has complex composition and high dynamics, and are often referred to as the second genome of plants (Berendsen et al., 2012). Studies have shown that soil-borne diseases are correlated with the number and activity of rhizosphere soil microbiota (Lee et al., 2021). Therefore, understanding the structure, function and interaction of rhizosphere soil microbiota (including the pathogens) is of great significance for the prevention and control of soil-borne diseases. At present, investigation on sugarcane rhizosphere soil microbiota is more on their effect on plant growth and agronomic traits, but very less about their contribution to root rot disease severity.

As the symptoms of sugarcane root rot is not prominent in the early stage, effective prevention methods are lacking (Tu., 2012). Traditional agricultural or chemical methods are used to control root rot, but are either time-consuming or limited by region, labor force and season in production and application (Liang et al., 2017). Besides, chemical control may cause problems including crop drug residues, microbial drug resistance, and/or soil quality decline or even degradation (Komárek et al., 2010). Biological control is an ideal alternative in controlling crop disease, due to its advantages of being friendly to the ecological environment, non-toxic or side effects to non-target organisms, and difficult for pathogens to develop drug resistance (Tariq et al., 2020). Studies have shown that plants can recruit antagonistic microorganisms to the pathogens, from the rhizosphere soil, which could be used as biocontrol agents to improve disease resistance (Mendes et al., 2011). Such biocontrol bacteria have the characteristics of fast reproduction speed, simple nutritional requirements, good environmental adaptability and strong root reproduction and colonization ability (Chen et al., 2020). A recent study found that *Bacillus* species had been isolated from banana rhizosphere soil, which can significantly promote the growth of banana seedlings and effectively prevent and control banana Fusarium wilt (Li et al., 2012). Biocontrol bacteria had been isolated from the rhizosphere soil of wheat and corn, which had strong inhibitory effects on soil-borne diseases caused by *Fusarium oxysporum*, *Sclerotinia sclerotiorum* and *Rhizoctonia solani* (Sun et al., 2014). *Pantoea agglomerans* produces and secretes Herbicolin A as a major antifungal compound to suppress Fusarium head blight (FHB) in cereals caused by *Fusarium graminearum* (Xu et al., 2022). Some biocontrol bacteria are also plant growth promoting rhizobacteria (PGPR). Such PGPR strains can directly or indirectly benefit host plants through their own metabolites or colonization advantages, such as improving plant disease resistance through nitrogen fixation, synthesis of siderophores and growth hormones (Du et al., 2016; Bloch et al., 2020). At present, the development of biocontrol bacteria had a good control effect on the root rot of medicinal plants (Mu et al., 2014), but the development of biocontrol bacteria in the rhizosphere soil of sugarcane root rot and the verification of the subsequent growth-promoting ability have not yet been reported.

With the aim to characterize rhizospheric microbes correlating/contributing to sugarcane root rot occurrence/severity, we collected roots and rhizosphere soil samples from the sugarcane displaying different severity of root rot disease, on a same sugarcane-planting plot in Guangzhou city, Guangdong Province, China. We categorize disease severity based on root phenotypes including total root length, total root surface area and total volume of the plants. We also characterized the composition and diversity of the rhizosphere soil microbial population correlating with different disease severity, using high-throughput sequencing technology. We further predicted rhizosphere soil microbiome function and generated a microbial co-occurrence network, for a better understanding of the relationship between the fungal pathogen *Fusarium* species and the bacterial community in the rhizosphere soil. Rhizospheric bacteria with biocontrol effects were isolated, identified, and their biocontrol ability was verified. Furthermore, some of the biocontrol bacteria displayed growth-promoting ability. Overall, our study depicts a correlation between rhizosphere microbial community and sugarcane root rot occurrence and/or severity, and providing candidate biocontrol/growth-promoting microbial resources with great potential in agricultural application.

## Materials and methods

### Sample collection and sugarcane root characterization

The sugarcane cultivars were planted in the same sugarcane plot in Nanshunyi Village, Dagang Town, Nansha District, Guangzhou City, Guangdong Province, China (22°47′04.4″N and 113°26′14.8″E), following regular irrigation, fertilization and maintenance practice. Samples used in this study were collected on 31 October 2019. We arbitrarily determined sugarcane root rot severity according to the height and stoutness of above-ground part of plants, as healthy (DHZA), mildly diseased (DHZB), moderately diseased (DHZC) and severely diseased (DHZD) groups (Supplementary Figure S1B). “DHZ” is short for “Dahuizhong”, the cultivar name called by the local farmer. Six sugarcane plantlets (adult-plants) from each group were randomly selected for collection of their roots and rhizosphere soil samples. The sampling follows the established method (Clemensson-Lindell and Persson., 1992): the roots of each sugarcane were dug out and loosely attached soil was removed by manual shaking, whereas the rhizosphere soil was collected from the surface of the roots. After collecting the rhizosphere soil, the sugarcane roots were washed with clean water, and air-dry. The cleaned root system and rhizosphere soil were sealed in sterile ziplock bags for later use. Photographs of the dried roots were taken, and total root length, root surface area, and root volume were measured using the software RhizoVision Explorer v1.0 (Seethepalli et al., 2021).

### Soil DNA extraction

Microbial DNA was extracted from fresh soil samples using the E. Z.N.A^®^ Soil DNA Kit (Omega Bio-Tek Inc., Norcross, GA, United States ). The concentration and purity of extracted DNA were measured using a NanoDrop 2000 spectrophotometer (Thermo Fisher Scientific, Waltham, MA, United States ). The microbial community structure was determined based on paired-end amplicon sequencing of the 16S rDNA gene (for bacteria) and the Internal Transcribed Spacer (ITS) region of fungal ribosomal DNA (for fungi) on the Illumina MiSeq PE 250 platform, following the established protocol (Schmidt et al., 2019). The V5-V7 hypervariable regions of the bacterial 16S rDNA gene were amplified using primers 799F and 1193R (Zhang et al., 2019) to assess bacterial communities, using the established PCR reaction conditions and procedures were described in Han et al. (2020). The fungal ITS1 region was amplified from each sample using primers ITS1F and ITS2R (Schmidt et al., 2019), which provided a comprehensive coverage with the highest taxonomical accuracy for fungal sequences, to assess fungal communities, using the reported PCR reaction conditions and procedures (Kang et al., 2021). PCR reaction was performed on a Bio-Rad S1000 Thermal Cycler (Bio-Rad, CA). Subsequently, high-throughput sequencing of the DNA libraries was performed using Illumina Miseq PE 250 platform and the sequences were deposited in the NCBI database with accession number PRJNA858005 and PRJNA858105 (http://www.ncbi.nlm.nih.gov), and GSA database (Chen et al., 2021) with accession number PRJCA011320 (https://ngdc.cncb.ac.cn/gsa/).

### Bioinformatics analysis

For multiple samples sequenced in parallel, the sliding window method was used to screen the quality of the paired-end sequences in the FASTQ format one by one, and then FLASH v1.2.11 (http://ccb.jhu.edu/software/FLASH/) was used to pair the paired-end sequences that passed the primary quality screening according to overlapping bases (Magoč and Salzberg., 2011). Subsequently, the ligated sequence identifications were assigned to corresponding samples, and QIIME2 v2020.2 (https://qiime2.org//) were used to demultiplexed raw sequences (Bolyen et al., 2019). Quality control functions in the DADA2 v1.5.2 (https://benjjneb.github.io/dada2/bigdata.html) plugin for precut, reverse reads and noise removal, chimera detection and removal to filter low quality reads, reconstruct amplicon sequence variants (ASVs), and generate feature tables for ASV counts (Callahan et al., 2016). Sequences belonged to archaea, mitochondria, and chloroplasts were removed. To avoid potential bias caused by differences in sequencing depth, the number of sequences in each sample was rarefied (Zhou et al., 2022). Bacterial and fungal community alpha diversity was processed in the Majorbio cloud (https://cloud.majorbio.com). Subsequently, at the ASV level, the microbial richness (Chao1) and diversity (Shannon) of rhizosphere soil microbiomes correlating with different disease severity (DHZA, DHZB, DHZC, and DHZD) were calculated to characterize the differences in alpha diversity between samples. Beta diversity is based on the Abundance-based Jaccard distance algorithm and the ANOSIM method, to evaluate and compare the differences between sample communities. Heatmaps, PCoA, and RDA were performed using the Majorbio cloud (http://cloud.majorbio.com).

### Function predicting of rhizosphere soil microbiome

BugBase (https://bugbase.cs.umn.edu/index.html) was used for prediction of bacterial phenotypes (Ward et al., 2017), including biofilm formation, pathogenicity, presence of mobile element(s), oxygen utilization (including aerobic, anaerobic, facultatively anaerobic, and oxidative stress tolerant), *etc.* The fungal community was analyzed by microecological guild using FUNGuild (Nguyen et al., 2016), which could be subdivided into three nutritional modes: pathotroph, symbiotroph and saprotroph. By respectively linking bacterial species classification and fungal species classification with phenotypic BugBase and functional FUNGuild classification, the classification of bacteria and fungi was predicted.

### Co-occurrence network analysis

The interaction between rhizosphere soil bacteria and the pathogen *Fusarium* genus was evaluated by determining the Spearman rank correlation coefficient and topological network properties of *Fusarium* and bacterial pairs. Co-occurrence networks were constructed through the MENA (molecular ecological network analysis pipeline) based analysis protocol (Feng et al., 2022). The network topology characteristics include the total number of network nodes (Operational Taxonomic Unit, OTU), total number of edges (connections between nodes), and degrees of co-occurrence (the number directly related to nodes) (Hartman et al., 2018). The co-occurrence network diagram was drawn using Cytoscape v.3.3.0 software (http://www.cytoscape.org/) to visualize the network.

### Isolation and identification of sugarcane root rot pathogen(s)

The diseased plants with typical symptoms of sugarcane root rot were collected and separated according to the tissue separation method (Xiao et al., 2020) as schematically presented in Supplementary Figure S1C. The fungal hyphae and spore were observed under a microscope, and classified according to *The Fusarium laboratory manual* (Leslie and Summerell., 2006), and verified whether they were pathogen according to Koch’s Law (Supplementary Figure S1D). For molecular identification, fungal genomic DNA was extracted using the fungal genomic DNA extraction kit (Omega Bio-Tek Inc., Norcross, GA, United States ) and subjected to PCR amplification using primers EF-1 and EF-2 (Geiser et al., 2004), following standard PCR reaction conditions and procedures (Herron 2015). The amplified products were sequenced by Sangon Biotechnology Co., Ltd. (Shanghai, China), followed by BLAST search (http://blast.ncbi.nlm.nih,gov/Blast.cgi) to identify the fungal genus. The phylogenetic analysis was performed with MEGA7 (https://www.megasoftware.net/).

### Screening and identification of biocontrol bacteria

After weighing 10 g of each rhizosphere soil sample, sterile water was used to prepare a soil dilution (Supplementary Figure S1E), which was then streaked to Nutrient Agar (NA) medium and cultured for 3 days. Single colonies with different colonial shapes and/or colors were isolated (Li et al., 2020), and evaluated by the confrontation culture method, the mycelial growth rate method and the spore germination method, following the established procedure (Li et al., 2020). The diameter of the fungal colony and the rate of fungal spore germination, with or without confrontation of screened bacterial strains, were measured, and used to calculate the inhibition rate using the following formula: mycelial growth inhibition (%) = (control colony diameter–treatment colony diameter)/(control colony diameter–inoculation plug diameter) ×100; spore germination inhibition (%) = (control spore germination rate–treatment spore germination rate)/control spore germination rate ×100.

All the selected biocontrol bacteria were identified to the species level based on their morphological characteristics, physiological and biochemical characteristics (Breed., 1984; Dong and Cai., 2001), and 16S rDNA sequence alignment. For PCR amplification of the bacterial 16S rDNA gene, universal primers 27F/1492R (Lane., 1991) were used. The amplified products were sequenced by Sangon Biotechnology Co., Ltd. (Shanghai, China) and subjected to BLAST search (http://blast.ncbi.nlm.nih,gov/Blast.cgi). Phylogenetic analysis was performed with MEGA7 (https://www.megasoftware.net/).

### Sugarcane root rot control effect and growth-promoting effect of biocontrol bacteria

The screened biocontrol bacteria were cultured in 100 ml of liquid Nutrient Broth (NB) medium, at 37°C, and shaking at 180 r/min, for 24 h. The bacterial cells were centrifuged and adjusted OD_600_ to 0.5 in sterile water. Such biocontrol bacteria suspension was evenly mixed with sterile soil and vermiculite in a ratio of 1:30, and placed in different U-PVC pipes, in which the healthy sugarcane tubers of equal size were transplanted, after disinfection (to avoid unequal residual microbes brought in by the tubers). The isolated pathogenic fungal strain (#3) that causes sugarcane root rot was cultured 100 ml of Potato Dextrose Broth (PDB) medium at 28°C and shanking at 180 r/min for 5 days, and diluted with sterile water to 1 × 10^6^ spores/ml. The fungal suspension was irrigated into the soil mixed with different biocontrol bacterial suspension, with the same proportion (Supplementary Figure S1F). Sugarcane grown in the soil containing no biocontrol bacteria served as the blank control. Three biological repeats were performed for each biocontrol bacteria treatment. After 45 days of sugarcane growth under different combination of fungal/bacterial inoculation, we dug out all the sugarcane roots, washed them with clean water, and air-dry, before imaging and measuring total root length, root surface area, and root volume using the software RhizoVision Explorer v1.0 (Seethepalli et al., 2021).

To assess the growth promoting effect, individual bacteria were inoculated to the sterile soils and vermiculite, following the same procedure as aforementioned. The sugarcane plants were allowed to grow in such bacterial inoculated soils for 45 days, before characterization of above-ground parts and roots. Qualitative determination of phosphorus-dissolving, nitrogen-fixing, siderophore and 3-indoleacetic acid capacities followed the established protocols (Syed-Ab-Rahman et al., 2018).

### Data analysis

All statistics analyses were performed using SPSS v21 software (IBM, United States ), and analyzed by Duncan test of analysis of variance (ANOVA). *p* values <0.05 were considered statistically significant.

## Results

### Phenotypic analysis of sugarcane root rot disease

**FIGURE 1:**
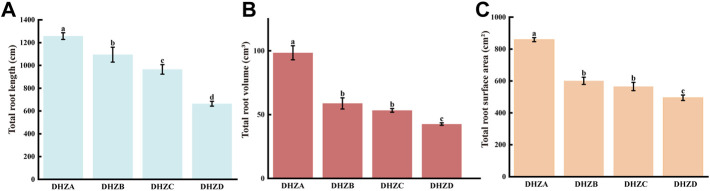
Categorization of sugarcane root rot severity based on total root length **(A)**, total root volume **(B)**, or total root surface area **(C)**. Samples were from DHZA: healthy plants, DHZB: mildly diseased plants, DHZC: moderately diseased plants, or DHZD: severely diseased plants. “DHZ” is short for “Dahuizhong”, the cultivar name called by the local farmer. Different lowercase letters denote significant differences at the *p <* 0.05 level by Duncan’s new multiple range test.

A longer root system, with a larger total surface area and volume, allows plants to absorb a wide range of soil water and nutrients, resulting in stronger plants (Deng et al., 2020). We arbitrarily determined sugarcane root rot severity, and categorized it as 4°: healthy (DHZA), mildly diseased (DHZB), moderately diseased (DHZC) and severely diseased (DHZD), based on the height and stoutness of above-ground part of sugarcane plantlets (Supplementary Figure S1B). “DHZ” is short for “Dahuizhong”, the cultivar name called by the local farmer. The total root length (Figure 1A), volume (Figure 1B) and surface area (Figure 1C) of sugarcane roots were significantly reduced, with increasing severity degrees. Compared with DHZA, the total root length of DHZB, DHZC or DHZD plantlets (adult-plants) decreased by 162.12, 292.46 and 593.95 cm, respectively; the total volume was decreased by 39.68, 45.15 and 55.88 cm^3^; and the total surface area was decreased by 258.02, 293.31 and 363.91 cm^2^, respectively. Overall, we confirmed that the sugarcane root rot severity degree was highly correlated to the growth displayed by the above-ground part of sugarcane.

### Microbial alpha diversity

**FIGURE 2:**
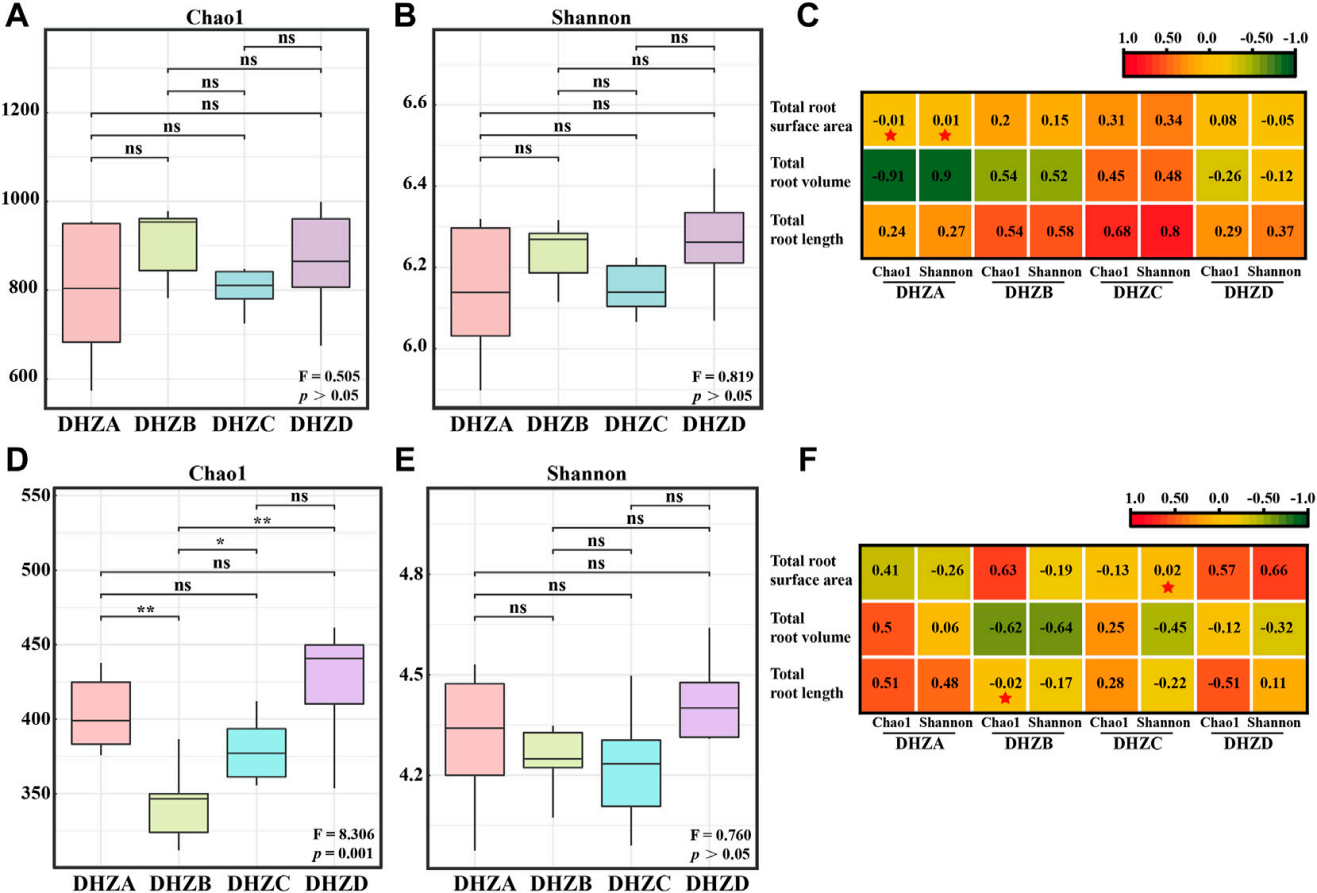
Bacterial and fungal alpha diversity analysis. Bacterial alpha-diversity represented by Chao 1 **(A)** or Shannon **(B)** indexes in rhizosphere soil samples from plants of different root rot severity. **(C)** Correlation between bacterial alpha-diversity and root phenotype factors based on Pearson analysis. Fungal alpha-diversity represented by Chao 1 **(D)** or Shannon **(E)** indexes in rhizosphere soil samples from plants of different root rot severity. For **(A)**, **(B)**, **(D)** and **(E)**, F, Fisher’s F-ratio; *p*, *p*-value. *p < 0.05, **p < 0.01. **(F)** Correlation between fungal alpha-diversity and root phenotype factors, using Pearson analysis. For **(C)** and **(F)**, *p < 0.05, **p < 0.01.

Alpha diversity showed no significant difference of rhizosphere soil bacterial community between different disease severity groups (*p* > 0.05, Figures 2A,B). Pearson correlation analysis showed that the total root surface area of DHZA was negatively correlated with the Chao1 index, but positively correlated with the Shannon index (Figure 2C). However, no significant correlation was found in other groups, between disease severity degrees and bacterial community diversity (Figure 2C). On the other hand, rhizosphere soil fungal community from DHZD group had the highest Chao1 and Shannon indices (Figures 2D,E). Particularly, the Chao1 index of rhizosphere soil fungal communities was significantly different among healthy and different diseased groups (*p* < 0.01, Figure 2D), while the Shannon index was not significantly different (*p* > 0.05, Figure 2E), indicating that the fungal community richness, but not the diversity, was correlating with disease severity. Pearson correlation analysis showed that the Chao1 index of the rhizosphere soil fungal community in DHZB group was negatively correlated with the total root length, and the Shannon index of the rhizosphere soil fungal community in DHZC group was positively correlated with the total root surface area (Figure 2F).

### Bacterial beta diversity and community composition

**FIGURE 3:**
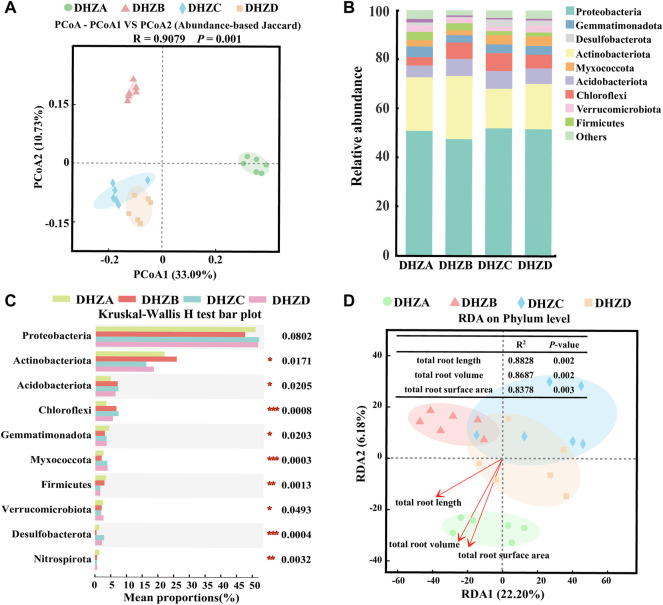
Bacterial beta diversity and microbiome composition analysis. **(A)** Principal coordinate analysis (PCoA) using the Abundance-based Jaccard distance matrix. **(B)** Relative abundance of different phyla in rhizosphere soil samples from plants of different sugarcane root rot severity. **(C)** Kruskal–Wallis H test analysis showing bacteria at the phylum level. *p < 0.05, **p < 0.01, ***p < 0.001. **(D)** Redundancy analysis (RDA) of rhizosphere bacterial community (dot) and root phenotype (arrows) indicates influential root phenotype factors.

PCoA analysis of the bacterial communities based on the Abundance-based Jaccard distance revealed that soil microbiome formed three distinct clusters (Figure 3A). The DHZA samples were separated from DHZB samples along the first axis (PERMANOVA, *p* < 0.05), while separated from DHZC and DHZD samples, which were clustered together (PERMANOVA, *p* < 0.05), along the second axis (PERMANOVA, *p* < 0.05) (Figure 3A). The microbiome in DHZA samples was mainly distributed in the positive value area, while those from DHZB, DHZC and DHZD samples in the negative value area, of PCoA1 (Figure 3A). These results confirm that sugarcane root rot disease significantly affected the rhizosphere bacterial community composition.

The bacterial community compositions varied significantly in different severity groups (Figure 3B). Proteobacteria and Actinobacteriota were most abundant in all sampled groups, accounting for more than 65% of the identified phyla. The rest of phylum level in the rhizosphere soil samples showed significant difference (*p* < 0.05) among groups DHZA-DHZD, except for Proteobateria, based on Kruskal–Wallis H test analysis (Figure 3C). The relative abundance of Actinobacteriota was decreased by 16.10% and 18.48% respectively in DHZC and DHZD, while increased by 3.91% in DHZB, as compared to that in DHZA. The trend of Proteobacteria abundance was opposite to that of Actinobacteriota, as it increased by 51.93% and 51.59% in DHZC and DHZD respectively, and decreased by 3.32% in DHZB, compared to that of DHZA. The relative abundances of Acidobacteriota and Chloroflexi were significantly increased in DHZB, DHZC and DHZD groups in comparison to healthy (DHZA) samples. In contrast, the relative abundance of Firmicutes decreased with increasing disease severity (Figures 3B,C). We further illustrated the correlation between disease severity degree and rhizosphere bacterial community composition by Redundancy Analysis (RDA) analysis. As shown in Figure 3D, RDA1 and RDA2 explained 22.20% and 6.18% of the sample differences, respectively. The composition of bacterial community was indeed affected by disease severity degree, which was reflected by the total root length (*R* ^2^ = 0.8828, *p* = 0.002), total root volume (*R* ^2^ = 0.8687, *p* = 0.002) and total root surface area (*R* ^2^ = 0.8378, *p* = 0.003). We speculate that the rhizosphere bacterial community composition was changed as a consequence of increased severity of disease.

### Fungal beta diversity and community composition

**FIGURE 4:**
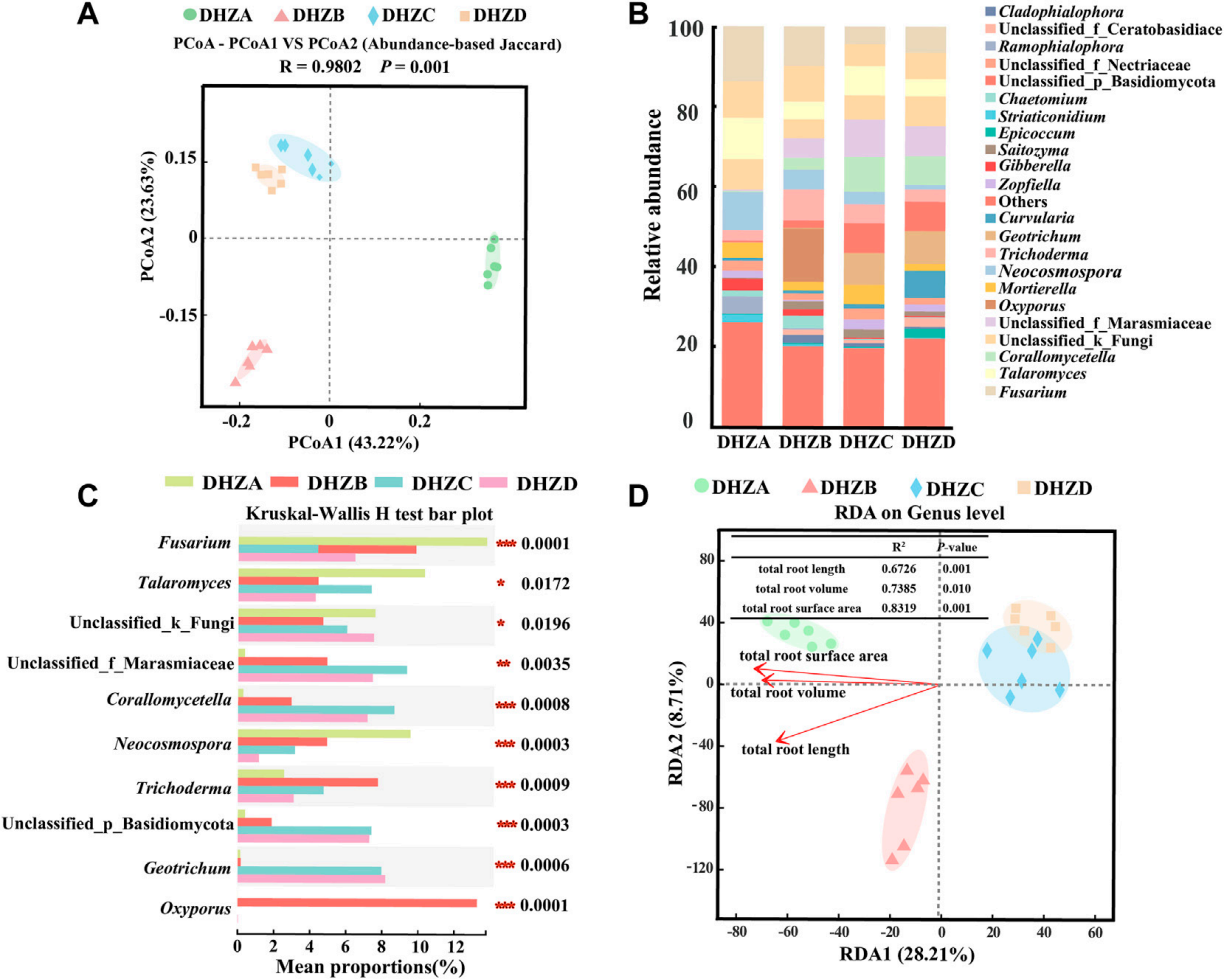
Fungal beta diversity and microbiome composition analysis. **(A)** PCoA using the Abund jaccard distance matrix, to analyze fungal species composition in different rhizosphere soil samples. **(B)** Relative abundance of different genus in different soil samples. **(C)** Kruskal–Wallis H test analyzed fungi at the genus level. *p < 0.05, **p < 0.01, ***p < 0.001. **(D)** RDA of rhizosphere fungal community (dot) and root phenotype (arrows) indicates influential root phenotype factors.

Similar as the bacterial communities, the fungal community diversity also formed three distinct clusters, based on PCoA analysis, with the rhizosphere soil fungal species composition of DHZC and DHZD groups in a cluster, and DHZA and DHZB respectively in a cluster (Figure 4A). The rhizosphere soil fungal microbiome of DHZA group was mainly distributed in the positive value area, while that of DHZB, DHZC and DHZD groups in the negative value area, of PCoA1 (Figure 4A). These results reveal significant change in the fungal species composition potentially resulted from disease progression.

We further assessed fungal relative abundances at the genus level, and found that *Fusarium*, *Talaromyces*, *Neocosmospora*, *Trichoderma* and *Gibberella* were commonly present in all the tested groups (Figure 4B). Furthermore, significant differences in the relative abundance of each genus were found among different groups (Figure 4C), supporting a correlation between fungal community composition and disease severity degree. The relative abundances of *Fusarium*, *Talaromyces*, and *Neocosmospora* in DHZA rhizosphere soil samples were 13.77%, 10.33%, and 9.56%, respectively, and totaled as 33.66% out of all identified fungal genera. Surprisingly, the totaled relative abundances of these genera decreased in DHZB, DHZC and DHZD rhizosphere soil samples, as 19.21%, 14.95% and 10.71% respectively. Notably, the relative abundances of *Fusarium*, the genus potentially containing the pathogen of sugarcane root rot as reported (Wang et al., 2018; Ren et al., 2021), was 13.77%, 9.85%, 4.41%, and 6.47% in group DHZA to DHZD, showing a negative correlation with increased disease severity (Figures 4B,C). RDA analysis confirmed that the composition of fungal community was also affected by disease severity degree, which was reflected by the total root length (*R* ^2^ = 0.6726, *p* = 0.001), total root volume (*R* ^2^ = 0.7385, *p* = 0.010) and total root surface area (*R* ^2^ = 0.8319, *p* = 0.001) (Figure 4D).

### Functional prediction of the rhizosphere soil microbiome

Phenotypic prediction of the rhizosphere soil bacterial microbiome was performed using BugBase database, and significant differences among DHZA to DHZD groups was found in terms of anaerobic, facultatively anaerobic, forming biofilms, potentially pathogenic, aerobic, and containing mobile elements, but not in stress tolerant, Gram positive and Gram negative (Supplementary Figure SA). It was worth noting that the predicted proportion of facultatively anaerobic and forming biofilms in rhizosphere soil bacteria was highest in the healthy (DHZA) group, as compared to diseased groups (Supplementary Figures S2B,C).

On the other hand, functional prediction of the rhizosphere soil fungal microbiome was performed using Fungi Functional Guild (FUNGuild) database. Saprotroph was the most common tropic type in all tested rhizosphere soil samples, with an average proportion of 46%, and the pathotroph in the second place, with an average proportion of 18.5%. It was worth noting that the proportion of saprotroph was higher, while pathotroph was lower, in DHZA group in comparison to DHZD group (Supplementary Figure S2D). Overall, from a biological point-of-view, we infer that decreased proportion of bacteria with facultatively anaerobic and forming biofilms functions, and of saprophytic fungi, accompanied with increased proportion of pathotropic fungi, could be viewed as an indicator of sugarcane root rot.

### Co-occurrence network analysis

**FIGURE 5:**
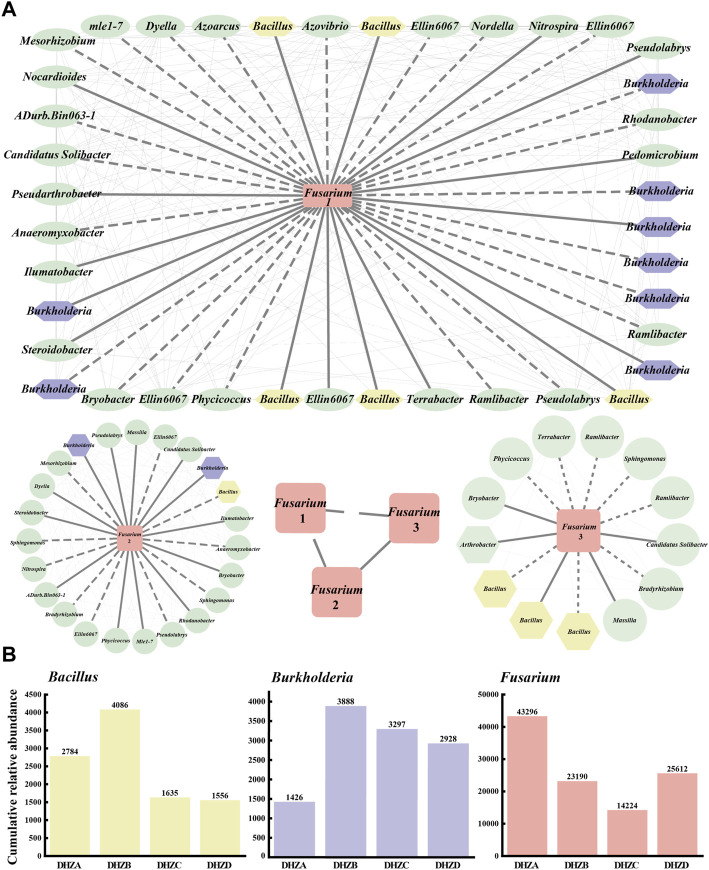
Co-occurrence networks and the relative abundance of selected microorganisms. **(A)** Co-occurrence networks visualizing significant correlations (ρ > 0.5 & ρ *<* −0.5, *p* < 0.05) between *Fusarium* pathogenic fungi and bacterial communities. The pathogenic fungus *Fusarium* (rectangle in the center of each network) was positively (solid lines) or negatively (dashed lines) connecting to the bacterial communities. Different *Fusarium* groups (*Fusarium* 1, *Fusarium* 2 and *Fusarium* 3). **(B)** The relative abundance of *Fusarium* pathogenic fungi and the top two correlated bacteria in different rhizosphere soil samples.

A co-occurrence network was constructed for a better understanding of the relationship between the fungal pathogen *Fusarium* species and the bacterial community in the rhizosphere soil. A connection with a magnitude >0.5 (positive correlation) or < -0.5 (negative correlation) and statistically significant (*p* < 0.05) was defined as correlation. The drawn network diagram was represented by different OTUs with positive and negative interactions, where the solid line for positive interaction indicates the change along the same trend among species, and the dashed line for negative interaction indicates the change along the opposite trend among species. We found correlations between different *Fusarium* groups (*Fusarium* 1, *Fusarium* 2 and *Fusarium* 3) and rhizosphere soil bacterial communities, among which *Burkholderia* had the largest number of species, with a total of 10, and *Bacillus* in the second place, with a total of 9 (Figure 5A). Correlation between three *Fusarium* groups also existed (Figure 5A). Notably, as the disease severity gradually increased, the cumulative relative abundances of *Fusarium*, *Burkholderia* and *Bacillus* species also accordingly changed, displaying an increase or decrease trend (Figure 5B). The DHZB group contained the most *Bacillus* and *Burkholderia* species, while the DHZA group contained the most *Fusarium* species in the rhizosphere soil (Figure 5B). Overall, our data demonstrate that a correlation between *Fusarium* species and rhizosphere soil bacterial communities, with *Burkholderia* and *Bacillus* species being more strongly correlated. Also, combined with the trend of cumulative relative abundance, it was speculated that some *Burkholderia* and *Bacillus* species may act as new antagonistic bacteria to inhibit the growth and reproduction of (pathogenic) *Fusarium* species. We infer that the plants infected by *Fusarium* species may tend to recruit beneficial microorganisms, including *Burkholderia* and *Bacillus* species, in order to suppress pathogenic microorganisms and/or boost plant immunity.

### Identification and verification of fungal pathogen and biocontrol bacteria from rhizospheric soil

Three fungal strain was isolated from the diseased samples, and named as GXUF-3. When grown on Potato Dextrose Agar (PDA) medium for 5 days, GXUF-3 formed luxuriant and white velvet-like mycelial colony, which was white when viewed from backside (Supplementary Figure S3A). CXUF-3 produced abundant large conidia of sickle- or spindle-shape, with 0–5 septum per conidium, when cultured in liquid carboxymethyl cellulose (CMC) medium (Supplementary Figure S3B). BLAST search using the PCR amplified Elongation factor 1-alpha (EF-1α) region of GXUF-3 showed a 100% homology with *Fusarium commune* (MF150040.1). The phylogenetic analysis based on the amplified EF-1α region showed that the GXUF-3 strain was clustered with several *F. commune* strains, and relatively far away from other *Fusarium* species (Supplementary Figure S3C). Overall, the GXUF-3 strain was identified as *F. commune.* To verify whether GXUF-3 strain was the causal pathogen of sugarcane root rot, we inoculated it in the sterilized soils that was used for planting sugarcane. After 45 days growth we observed that the infected sugarcane roots were seriously hindered, gradually turning brown, soft and rotten, and meanwhile the above-ground part was dwarfed, and the leaves turned yellow (Supplementary Figure S3D). In contrast, the plants inoculated with sterile water (untreated control) grew normally and showed no disease symptoms (Supplementary Figure S3D). The re-isolated fungal strain from the infected sugarcane displayed similar colonial morphology as GXUF-3 (Supplementary Figure S3D), confirming that GXUF-3 was the causal pathogen of sugarcane root rot.

A total of 273 bacterial strains were isolated from the tested rhizosphere soil samples, among which 7 strains with potential biocontrol effects were obtained by confrontation culture with *F. commune* strain GXUF-3. We named these seven biocontrol strains as GXUB-1, GXUB-2, GXUB-3, GXUB-4, GXUL-1, GXUL-2, GXUJ-1 (Supplementary Figure S4A). GXUL-1 strain displayed the weakest, while GXUB-1 strain with the strongest, inhibitory effect on fungal mycelial growth (Supplementary Figures S4A,B). Furthermore, we found that mycelial growth and spore germination of GXUF-3 was suppressed by different concentration of biocontrol bacterial fermentation broth (Supplementary Figures S4C,D), suggesting that the tested bacterial strains produced anti-fungus compound(s). We also noticed a significant difference in mycelial growth and spore germination inhibition rates among different bacteria strains (Supplementary Figures S4C, D; Supplementary Table S1). Overall, we obtained seven bacterial strains with varied inhibitory effect on mycelial growth and spore germination of the fungal pathogen causing sugarcane root rot.

**FIGURE 6:**
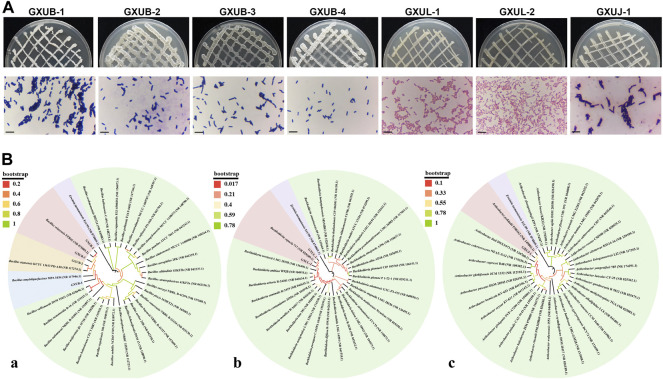
Identification of 7 isolated biocontrol bacteria. **(A)** Colony morphology and Gram staining of different biocontrol bacteria. Scale bar = 50 µm. **(B)** Phylogenetic analysis of biocontrol bacteria based on 16S rDNA sequences. **(A)** *Bacillus* species; **(B)** *Burkholderia* species; **(C)** *Arthrobacter* species.

Based on morphological (Figure 6A), physiological and biochemical characterization (Supplementary Table S2), and molecular identification (Figure 6B), we identified GXUB-1 and GXUB-3 as *B. velezensis*, GXUB-2 as *B. siamensis*, and GXUB-4 as *B. amyloliquefaciens*; GXUL-1 and GXUL-2 as *B. cepacia,* and GXUJ-1 as *A. pokkalii*.

**FIGURE 7:**
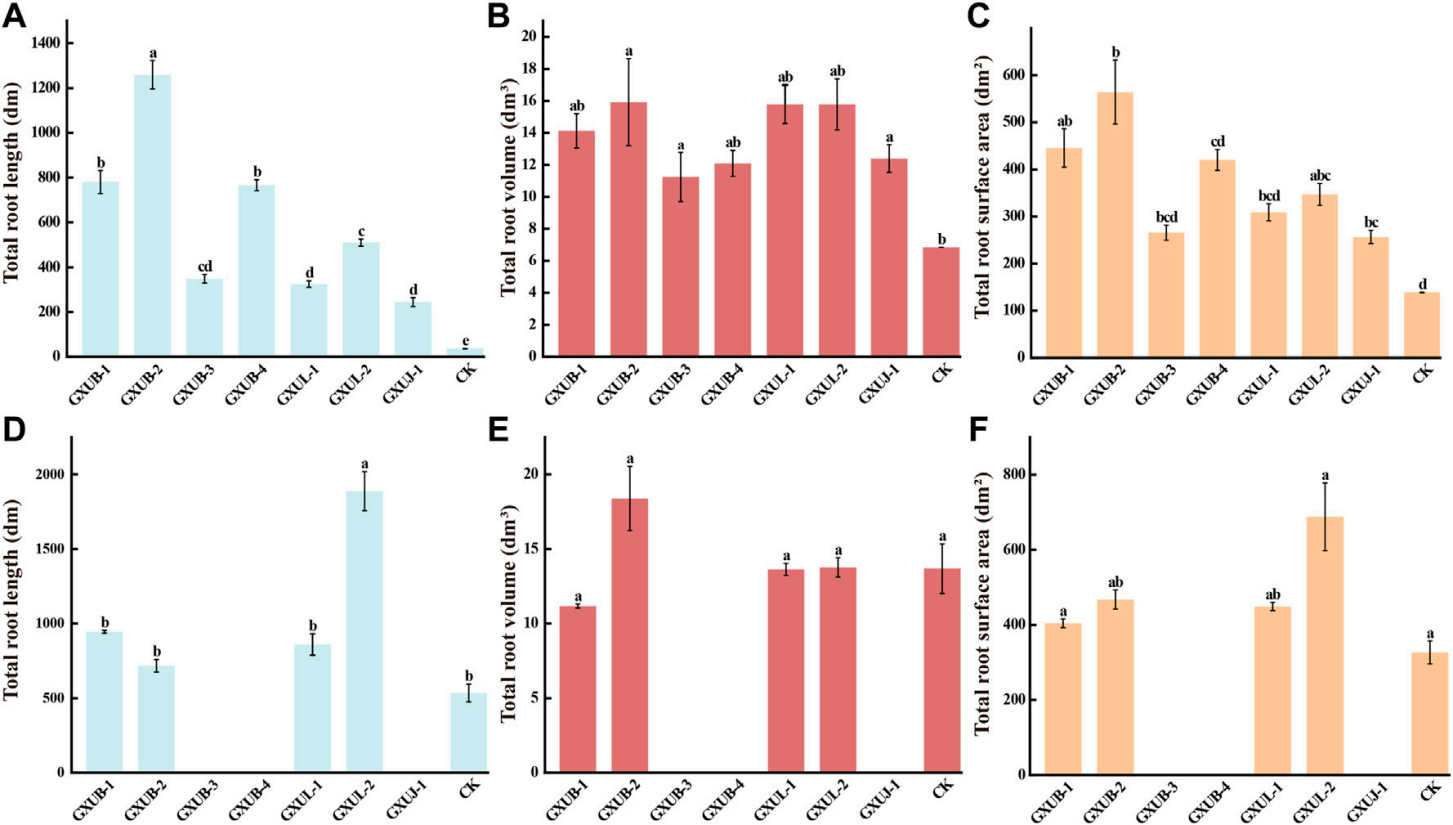
Disease control and growth-promoting effect of biocontrol bacteria strains.Quantification of disease control effects of different biocontrol bacteria on root phenotypes of sugarcane root rot based on total root length **(A)**, total root volume **(B)**, or total root surface area **(C)**. Quantification of growth-promoting effects of different biocontrol bacteria based on root phenotypes of sugarcane root rot based on total root length **(D)**, total root volume **(E)**, or total root surface area **(F)**. Different lowercase letters denote significant differences at the *p <* 0.05 level by Duncan’s new multiple range test.

To test control effect of these seven strains on sugarcane root rot, we inoculated them individually, and respectively with fungal pathogen, to the sterile soils, on which sugarcane was planted. After 45 days growth, the total root length, total root surface area and total root volume of sugarcane were measured. Obvious changes of root phenotypes were seen in the biocontrol bacteria treated seedling, as compared to the control group which was inoculated with fungal pathogen alone (Figures 7A–C), confirming that all the tested biocontrol bacteria were effective in suppressing sugarcane root rot, among which *B. siamensis* GXUB-2 strain had the best control effect (Figures 7A–C).

To test the growth-promoting property of these seven strains, sugarcane was planted in bacteria inoculated soils, without fungal pathogen, for 45 days, and the root phenotypes were characterized. Obvious differences were visualized in the root phenotypes of sugarcane under different bacterial inoculation (Figures 7D–F). Surprisingly, inoculation with GXUB-3, GXUB-4 or GXDJ-1 alone suppressed growth of roots part (Figures 7D–F), while in presence of fungal pathogen, all these three strains displayed disease suppressing property (Figures 7A–C). In contrast, inoculation with the other four strains caused increase in the total root length and total root surface ar.

The ability of nitrogen fixation, phosphorus solubilization, phytohormea (Figures 7D–F). GXUB-1 and GXUL-1 caused a slight decrease, while GXUB-2 and GXUL-2 caused increase, in the total root volume (Figures 7D–F). In summary, we found that *B. velezensis* GXUB-3 strain, *B. amyloliquefaciens* GXUB-4 and *A. pokkalii* GXUJ-1 strain had a growth-inhibitory effect on the sugarcane roots, and the other four strains with growth-promoting property, among which *B. siamensis* GXUB-2 and *B. cepacia* GXUL-2 strains behaved better (Figures 7D–F)one(s) production, and siderophore production were assessed in these seven strains. Both GXUL-1 and GXUL-2 strains were able to produce the phytohormone indole-3-acetic acid (IAA), siderophores, and able to fix nitrogen and solubilize phosphorus (Supplementary Table S3). GXUB-1 displayed only siderophores producing ability (Supplementary Table S3). The three strains with inhibitory effect on sugarcane growth, GXUB-3, GXUB-4 and GXUJ-1, possessed none of the tested activities. However, GXUB-2 strain with the best disease suppression effect and an ideal growth-promoting effect, also did not possess four tested properties (Supplementary Table S3), suggesting that its biocontrol and growth-promoting mechanism may lie in other untested aspects.

## Discussion

In this study, we isolated and verified a *F. commune* strain (GXUF-3) as the causal pathogen of sugarcane root rot, as consistent with a previous report (Wang et al., 2018). We further found that sugarcane root rot led to the destruction of sugarcane roots, reflected by the root phenotypes including reduction in total root length, total root surface, and total volume (Figure 1). Such change in root morphology is likely result in weakened root function, and thus led to dwarf above-ground part, and thus seriously affected the growth of sugarcane ratoons and the economics of sugarcane production (Pissolato et al., 2021). By microbiome analysis, we found that rhizosphere soil bacterial community diversity was not significantly changed among different severity degree of sugarcane root rot (Figures 2A,B), which is similar with a previous report on the avocado root rot (Solís-García et al., 2021). However, we found significant differences in the fungal community richness between rhizosphere soil from diseased and healthy plants (Figure 2D). We infer that the pathogen *Fusarium* species may have an advantage in interspecific competition, and therefore become dominant species in the community during disease progress. Such phenomenon was also reported in *Fusarium* root rot disease to other crops (Wang et al., 2017). We further found an obvious correlation between disease severity and microbial community structure in the rhizosphere soil, based on PCoA (Figure 3A, Figure 4A), Kruskal–Wallis H test (Figure 3C, Figure 4C), and RDA (Figure 3D, Figure 4D) analyses. A higher relative abundance of Firmicutes in rhizosphere soil from healthy plant than diseased plant (Figure 3B) suggests that this phylum may play a role in disease suppression. Consistent with this observation, *Bacillus* species belonging to Firmicutes, were in a strong co-occurrence interaction network with *Fusarium* pathogen(s) (Figure 5A). In terms of fungal microbiome in the rhizosphere soil, we found that *Fusarium*, *Talaromyces* and *Neocosmospora* were dominant in healthy group (>30%), while declined in diseased groups (< 20%; Figure 4B). We infer that it may at least partially be due to the significant bacteriostatic effect of *Talaromyces* on common soil-borne disease pathogens, as reported (Tian et al., 2021). BugBase and FUNGuild were respectively used to predict bacterial and fungal function in the tested rhizosphere soil samples. We found facultatively anaerobic and biofilms forming bacteria were significantly higher in healthy group than in disease groups (Supplementary Figures S2B and C). On the other hand, we found saprotrophic fungi occupied a higher proportion in the healthy group, while pathotropic fungi increased in the diseased groups (Supplementary Figure S2D). Co-occurrence network analysis can be used to reveal highly related taxa in the community, for a better understanding of the association between bacteria and fungi (Zheng et al., 2021; Jin et al., 2022). In this study, we demonstrated the *Bacillus* and *Burkholderia* were in a strong co-occurrence interaction network with *Fusarium* pathogen(s) (Figure 5A), and 6 out of 7 isolated bio-control bacterial strains were identified as *Bacillus* and *Burkholderia* species (Figure 6) from the rhizosphere soil. Additional, the observed accumulation of *Burkholderia* and *Bacillus*, and the concomitant decline of *Fusarium*, in rhizosphere soil with increased disease severity (Figure 5B), is possibly due to the recruitment of beneficial microorganisms in response to *Fusarium* infection to the host cane. This observation is consistent with the report that the stressed plants tend to recruit beneficial microorganisms, to suppress pathogenic microorganisms (Liu et al., 2021). Alternatively, the apparent negative correlation between the relative abundances of *Fusarium* and disease severity may resulted from increased fungal community richness, particularly increase of saprotrophs, as leak of nutrients from rot roots to rhizosphere soil along with increase disease severity. To our knowledge, our study is the first report on the relationship between the root phenotype of the sugarcane root rot disease, and rhizosphere soil microbiome composition and function.

Biocontrol bacteria are ideal alternatives of crop disease control, by producing biologically active metabolites such as antibiotics, bacteriocins and siderophores (Gajbhiye and Kapadnis., 2016). In this study, we identified *Bacillus* and *Burkholderia* as the bacterial groups that were strongly related to the *Fusarium* pathogens of sugarcane root rot (Figure 5A). We further isolated 7 biocontrol bacteria strains, including species of *Bacillus*, *Burkholderia* and *Arthrobacter* (Figure 6), with strong suppression effect on *Fusarium* pathogen mycelial growth and spore germination (Supplementary Figure S4) and disease occurrence (Figures 7A–C). Among them, *Bacillus* strains displayed the strongest ability on disease control (Figures 7A–C). As a major source of biocontrol agents, *Bacillus* genus possesses numerous merits including eco-friendly, inexpensive and sustainable (Chen Y. S. et al., 2020). Application of *Bacillus* strain in controlling root rot disease has been reported in *Astragalus* (Gao et al., 2019), a*nd Burkholderia* strain has also been reported to control root rot in maize (Tagele et al., 2019)*.* On the other hand, *Arthrobacter* strain was reported in management of *Fusarium* diseases (Barrows-Broaddus et al., 1985). Our study, for the first time, shows the potential of *Bacillus*, *Burkholderia* and *Arthrobacter* in biocontrol of sugarcane root rot. Among them, *Bacillus* and *Burkholderia* strains were found to possess grow-promoting property (Figures 7D–F), part of which may lie in their ability of nitrogen fixation, phosphorus solubilization, phytohormone or siderophore production (Supplementary Table S1D). Such growth-promoting mechanisms have been reported in various *Burkholderia* species (Elliott et al., 2007; Ghosh et al., 2019). Furthermore, it is worth testing the effect of mixing bacterial strains with best disease inhibitory effect, and those with best growth-promoting effect, in future.

In summary, we characterized the relationship between rhizospheric soil microbes and sugarcane root rot occurrence/severity, which provided a theoretical basis for the prevention and control of sugarcane root rot by micro-ecological regulation. Besides rhizosphere soil microbiome, other potential factors including environmental variables, sugarcane genotype, soil edaphic factors, may also contribute to the crop disease, and affect each other, which awaits further (Wang et al., 2017) investigation in future.

## Data Availability

The datasets presented in this study can be found in online repositories. The names of the repository/repositories and accession number(s) can be found in the article/Supplementary Material.

## References

[B1] Barrows-Broaddus J. Dwinell L. D. Kerr T. J. (1985). Evaluation of *Arthrobacter* sp. for biological control of the pitch canker fungus (*Fusarium moniliforme* var. *subglutinans*) on slash pines. Can. J. Microbiol. 31, 888–892. 10.1139/m85-166

[B2] Berendsen R. L. Pieterse C. M. J. Bakker P. A. H. M. (2012). The rhizosphere microbiome and plant health. Trends Plant Sci. 17, 478–486. 10.1016/j.tplants.2012.04.001 22564542

[B3] Bloch S. E. Clark R. Gottlieb S. S. Wood L. K. Shah N. Mak S. (2020). Biological nitrogen fixation in maize: Optimizing nitrogenase expression in a root-associated diazotroph. J. Exp. Bot. 71, 4591–4603. 10.1093/jxb/eraa176 32267497 PMC7382387

[B4] Boaretto L. F. Labata M. T. V. Franceschini L. M. Cataldi T. R. Budzinski I. G. F. Moraes F. E. (2021). Proteomics reveals an increase in the abundance of glycolytic and ethanolic fermentation enzymes in developing sugarcane culms during sucrose accumulation. Front. Plant Sci. 12, 716964. 10.3389/fpls.2021.716964 34659289 PMC8515036

[B5] Bolyen E. Rideout J. R. Dillon M. R. Bokulich N. A. Abnet C. C. Al-Ghalith G. A. (2019). Reproducible, interactive, scalable and extensible microbiome data science using QIIME 2. Nat. Biotechnol. 37, 852–857. 10.1038/s41587-019-0209-9 31341288 PMC7015180

[B6] Breed (1984). Bergey’s manual of systematic bacteriology. USA: Maryland Baltimote Williams & Wilkins Press. 36-37 & 63-65 & 172-178.

[B7] Callahan B. J. McMurdie P. J. Rosen M. J. Han A. W. Johnson A. J. A. Holmes S. P. (2016). DADA2: High-resolution sample inference from Illumina amplicon data. Nat. Methods 13, 581–583. 10.1038/nmeth.3869 27214047 PMC4927377

[B8] Chen K. Tian Z. H. He H. Long C. A. Jiang F. T. (2020). *Bacillus* species as potential biocontrol agents against citrus diseases. Biol. Control 151, 104419. 10.1016/j.biocontrol.2020.104419

[B9] Chen T. T. Chen X. Zhang S. S. Zhu J. W. Tang B. X. Wang A. K. (2021). The genome sequence archive family: Toward explosive data growth and diverse data types. Genomics Proteomics Bioinforma. 19, 578–583. 10.1016/j.gpb.2021.08.001 PMC9039563 34400360

[B10] Chen Y. S. Yin Q. Sang Z. Y. Zhu Z. L. Duan J. Ma L. Y. (2020). Research progress in occurrence and control measure of tree root rot. World For. Res. 33, 26–32. 10.13348/j.cnki.sjlyyj.2019.0091.y

[B11] Clemensson-Lindell A. Persson H. (1992). Effects of freezing on rhizosphere and root nutrient content using two soil sampling methods. Plant Soil 139, 39–45. 10.1007/bf00012840

[B12] Deng J. Feng X. Q. Wang D. Y. Lu J. Chong H. T. Shang C. (2020). Root morphological traits and distribution in direct-seeded rice under dense planting with reduced nitrogen. PLoS One 15, e0238362. 10.1371/journal.pone.0238362 32877452 PMC7467324

[B13] Dong X. Cai M. Y. (2001). Handbook of systematic identification of common bacteria. China: Science Press, 62–67. & 162-171 & 272.

[B14] Du N. S. Li S. Yuan Y. H. Li B. Shu S. Sun J. (2016). Proteomic analysis reveals the positive roles of the plant-growth-promoting rhizobacterium NSY50 in the response of cucumber roots to *Fusarium oxysporum* f. sp. *cucumerinum* inoculation. Front. Plant Sci. 7, 1859. 10.3389/fpls.2016.01859 28018395 PMC5155491

[B15] Elliott G. N. Chen W. M. Chou J. H. Wang H. C. Sheu S. Y. Perin L. (2007). *Burkholderia phymatum* is a highly effective nitrogen-fixing symbiont of *Mimosa* spp. and fixes nitrogen *ex planta* . New Phytol. 173, 168–180. 10.1111/j.1469-8137.2006.01894.x 17176403

[B16] Feng K. Peng X. Zhang Z. Gu S. S. He Q. Shen W. S. (2022). iNAP: an integrated network analysis pipeline for microbiome studies. iMeta 1, e13. 10.1002/imt2.13 PMC10989900 38868563

[B17] Gajbhiye M. H. Kapadnis B. P. (2016). Antifungal-activity-producing lactic acid bacteria as biocontrol agents in plants. Biocontrol Sci. Technol. 26, 1451–1470. 10.1080/09583157.2016.1213793

[B18] Gao F. Zhao X. X. Yan H. Lei Z. H. Wang M. L. Qin X. M. (2019). Screening and identification of antagonistic *Bacillus* against *Astragalus membranaceus* root rot and its effect on microorganism community in root zone soil. Chin. J. Chin. Mater Med. 44, 3942–3947. 10.19540/j.cnki.cjcmm.20190701.108 31872728

[B19] Geiser D. M. Jiménez-Gasco M. M. Kang S. Makalowska I. Veeraraghavan N. Ward T. J. (2004). FUSARIUM-ID v. 1.0: A DNA sequence database for identifying *Fusarium* . Eur. J. Plant Pathology 110, 473–479. 10.1023/b:ejpp.0000032386.75915.a0

[B20] Ghosh R. Barman S. Mandal N. C. (2019). Phosphate deficiency induced biofilm formation of *Burkholderia* on insoluble phosphate granules plays a pivotal role for maximum release of soluble phosphate. Sci. Rep. 9, 5477. 10.1038/s41598-019-41726-9 30940828 PMC6445130

[B21] Han Q. Ma Q. Chen Y. Tian B. Xu L. X. Bai Y. (2020). Variation in rhizosphere microbial communities and its association with the symbiotic efficiency of rhizobia in soybean. ISME J. 14, 1915–1928. 10.1038/s41396-020-0648-9 32336748 PMC7367843

[B22] Hartman K. van der Heijden M. G. A. Wittwer R. A. Banerjee S. Walser J. Schlaeppi K. (2018). Cropping practices manipulate abundance patterns of root and soil microbiome members paving the way to smart farming. Microbiome 6, 14. 10.1186/s40168-017-0389-9 29338764 PMC5771023

[B23] Herron D. A. Wingfield M. J. Wingfield B. D. Rodas C. A. Marincowitz S. Steenkamp E. T. (2015). Novel taxa in the *Fusarium fujikuroi* species complex from *Pinus* spp. Stud. Mycol. 80, 131–150. 10.1016/j.simyco.2014.12.001 26955193 PMC4779798

[B24] Jin X. Wang Z. L. Wu F. Z. Li X. G. Zhou X. G. (2022). Litter mixing alters microbial decomposer community to accelerate tomato root litter decomposition. Microbiol. Spectr. 10, e0018622. 10.1128/spectrum.00186-22 35604181 PMC9241821

[B25] Kang L. He D. M. Wang H. Han G. Q. Lv H. Y. Xiao W. T. (2021). Breeding on mountains'' resulted in the reorganization of endophytic fungi in asexually propagated plants (*Ligusticum chuanxiong* Hort.). Front. Plant Sci. 12, 740456. 10.3389/fpls.2021.740456 34858448 PMC8631752

[B26] Komárek M. Čadková E. Chrastný V. Bordas F. Bollinger J. (2010). Contamination of vineyard soils with fungicides: A review of environmental and toxicological aspects. Environ. Int. 36, 138–151. 10.1016/j.envint.2009.10.005 19913914

[B27] Lane D. J. (1991). “16S/23S rRNA sequencing,” in Nucleic acid techniques in bacterial systematics. Editors Stackebrandt E. Goodfellow M. (New York: John Wiley & Sons), 115–117.

[B28] Lee S. Kong H. G. Song G. C. Ryu C. (2021). Disruption of Firmicutes and Actinobacteria abundance in tomato rhizosphere causes the incidence of bacterial wilt disease. ISME J. 15, 330–347. 10.1038/s41396-020-00785-x 33028974 PMC7852523

[B29] Leslie J. F. Summerell B. A. (2006). The *Fusarium* laboratory manual. Hoboken, NJ, USA: Blackwell Press, 113–117.

[B30] Li J. Wei S. H. Xu Q. Y. Kong L. C. Yang M. R. Wang Y. L. (2020). Screening and identification of antagonistic bacteria against rice blast smut. Agrochemicals 59, 676–679. CNKI:SUN:NYZZ.0.2020-09-013.

[B31] Li W. Y. Peng Z. P. Yang S. H. Yu J. H. Huang J. C. Wu X. N. (2012). Effect of plant growth-promoting rhizobacteria on growth and controlling Fusarium-wilt disease of banana seedlings. Acta Horiticulturae Sin. 39, 234–242. CNKI:SUN:YYXB.0.2012-02-007.

[B32] Liang X. T. Xu J. (2017). Application analysis of MATLAB image processing technology in the identification of agricultural diseases and insect pests. South China Agri 11, 117–124. 10.19415/j.cnki.1673-890x.2017.21.065

[B33] Liu H. W. Li J. Y. Carvalhais L. C. Percy C. D. Verma P. J. Schenk P. M. (2021). Evidence for the plant recruitment of beneficial microbes to suppress soil-borne pathogens. New Phytol. 229, 2873–2885. 10.1111/nph.17057 33131088

[B34] Magoč T. Salzberg S. L. (2011). Flash: Fast length adjustment of short reads to improve genome assemblies. Bioinformatics 27, 2957–2963. 10.1093/bioinformatics/btr507 21903629 PMC3198573

[B35] Mendes R. Garbeva P. Raaijmakers J. M. (2013). The rhizosphere microbiome: Significance of plant-beneficial, plant-pathogenic and human-pathogenic microorganisms. FEMS Microbiol. Rev. 37, 634–663. 10.1111/1574-6976.12028 23790204

[B36] Mendes R. Kruijt M. de Brujin I. Dekkers E. van der Voort M. Schneider J. H. M. (2011). Deciphering the rhizosphere microbiome for disease-suppressive bacteria. Science 332, 1097–1100. 10.1126/science.1203980 21551032

[B37] Mu X. R. Ma Y. Y. Yang Z. Z. Ma L. Jiang Y. B. (2014). Research advance on the control of root rot disease of medical plants. Pharm. Clin. Chin. Materia Medica 5, 5–8. CNKI:SUN:LCZY.0.2014-02-002.

[B38] Nguyen N. H. Song Z. Bates S. T. Baranco S. Tedersoo L. Menke J. (2016). FUNGuild: An open annotation tool for parsing fungal community data sets by ecological guild. Fungal Ecol. 20, 241–248. 10.1016/j.funeco.2015.06.006

[B39] Pang Z. Q. Tayyab M. Kong C. B. Liu Q. Liu Y. M. Hu C. H. (2021). Continuous sugarcane planting negatively impacts soil microbial community structure, soil fertility, and sugarcane agronomic parameters. Microorganisms 9, 2008. 10.3390/microorganisms9102008 34683329 PMC8537732

[B40] Peiffer J. A. Spor A. Koren O. Jin Z. Tringe S. G. Dangl J. L. (2013). Diversity and heritability of the maize rhizosphere microbiome under field conditions. Proc. Natl. Acad. Sci. U. S. A. 110, 6548–6553. 10.1073/pnas.1302837110 23576752 PMC3631645

[B41] Pissolato M. D. da Cruz L. P. Silveira N. M. Machado E. C. Ribeiro R. V. (2021). Sugarcane regrowth is dependent on root system size: An approach using young plants grown in nutrient solution. Bragantia 80, e4321. 10.1590/1678-4499.20210039

[B42] Potocka I. Szymanowska-Pulka J. (2018). Morphological responses of plant roots to mechanical stress. Ann. Bot. 122, 711–723. 10.1093/aob/mcy010 29471488 PMC6215033

[B43] Qu Y. K. Tang J. Liu B. Lyu H. Duan Y. C. Yang Y. (2022). Rhizosphere enzyme activities and microorganisms drive the transformation of organic and inorganic carbon in saline-alkali soil region. Sci. Rep. 12, 1314. 10.1038/s41598-022-05218-7 35079055 PMC8789911

[B44] Ren Q. X. Zhang J. X. Wang J. H. Xu S. Q. Yao W. Zhang M. Q. (2022). Isolation, identification, and biological characteristics analysis of pathogenic fungi causing root rot disease of chewing cane. Shandong Agric. Sci. 54, 135–142. 10.14083/j.issn.1001-4942.2022.07.019

[B45] Ren Q. X. Zhang J. X. Zhang M. Q. (2021). Research progress on sugarcane root rot and its pathogenic *Fusarium commune* . Sugarcane Canesugar 50, 49–57. 10.3969/j.issn.1005-9695.2021.03.011

[B46] Schmidt J. E. Kent A. D. Brisson V. L. Gaudin A. C. M. (2019). Agricultural management and plant selection interactively affect rhizosphere microbial community structure and nitrogen cycling. Microbiome 7, 146. 10.1186/s40168-019-0756-9 31699148 PMC6839119

[B47] Seethepalli A. Dhakal K. Griffiths M. Guo H. C. Freschet G. T. York L. M. (2021). RhizoVision explorer: Open-source software for root image analysis and measurement standardization. AoB Plants 13, plab056. 10.1093/aobpla/plab056 34804466 PMC8598384

[B48] Solís-García I. A. Ceballos-Luna O. Cortazar-Murillo E. M. Desgarennes D. Garay-Serrano E. Patiño-Conde V. (2021). Phytophthora root rot modifies the composition of the avocado rhizosphere microbiome and increases the abundance of opportunistic fungal pathogens. Front. Microbiol. 11, 574110. 10.3389/fmicb.2020.574110 33510714 PMC7835518

[B49] Sun G. Z. Yao T. Liu T. Lu H. (2014). Antagonism of plant growth promoting rhizobacteria on three soil-borne fungous pathogens. Microbiol. China 41, 2293–2300. 10.13344/j.microbiol.china.140524

[B50] Syed-Ab-Rahman S. F. Carvalhais L. C. Chua E. Xiao Y. W. Wass T. Schenk P. M. (2018). Identification of soil bacterial isolates suppressing different *Phytophthora* spp. and promoting plant growth. Front. Plant Sci. 9, 1502. 10.3389/fpls.2018.01502 30405657 PMC6201231

[B51] Tagele S. B. Kim S. W. Lee H. G. Lee Y. S. (2019). Potential of novel sequence type of *Burkholderia cenocepacia* for biological control of root rot of maize (*Zea mays* L.) caused by *Fusarium temperatum* . Int. J. Mol. Sci. 20, 1005. 10.3390/ijms20051005 30813526 PMC6429479

[B52] Tariq M. Khan A. Asif M. Khan F. Ansari T. Shariq M. (2020). Biological control: A sustainable and practical approach for plant disease management. Acta Agric. Scand. Sect. B —. Soil & Plant Sci. 70, 507–524. 10.1080/09064710.2020.1784262

[B53] Tian Y. H. Zhao Y. Fu X. S. Yu C. M. Gao K. X. Liu H. X. (2021). Isolation and identification of *Talaromyces* sp. strain Q2 and its biocontrol mechanisms involved in the control of *Fusarium* wilt. Front. Microbiol. 12, 724842. 10.3389/fmicb.2021.724842 34690965 PMC8531730

[B54] Tu Y. (2012). Research progress on main root diseases of fruit trees and their control methods. Jiangsu Agric. Sci. 40, 132–134. 10.3969/j.issn.1002-1302.2012.10.047

[B55] Vives-Peris V. López-Climent M. F. Pérez-Clemente R. M. Gómez-Cadenas A. (2020). Root involvement in plant responses to adverse environmental conditions. Agronomy 10, 942. 10.3390/agronomy10070942

[B56] Wang J. H. Chai Z. Bao Y. X. Wang H. X. Li Y. S. Rao G. P. (2018). First report of *Fusarium commune* causing root rot disease of sugarcane (var. Badila) in China. Plant Dis. 102, 1660–1664. 10.1094/PDIS-07-17-1011-PDN

[B57] Wang M. Chen M. N. Yang Z. Chen N. Chi X. Y. Pan L. J. (2017). Influence of peanut cultivars and environmental conditions on the diversity and community composition of pod rot soil fungi in China. Mycobiology 45, 392–400. 10.5941/myco.2017.45.4.392 29371808 PMC5780372

[B58] Wang R. Zhang H. C. Sun L. G. Qi G. F. Chen S. Zhao X. Y. (2017). Microbial community composition is related to soil biological and chemical properties and bacterial wilt outbreak. Sci. Rep. 7, 343. 10.1038/s41598-017-00472-6 28336973 PMC5428506

[B59] Ward T. Larson J. Meulemans J. Hillmann B. Lynch J. Sidiropoulos D. (2017). BugBase predicts organism level microbiome phenotypes. bioRxiv, 1–19. 10.1101/133462

[B60] Xiao R. F. Chen Y. P. Chen M. C. Ruan C. Q. Zhu Y. J. Liu B. (2020). Pathogen identification of root rot of *Pseudostellaria heterophylla* plant and fungicide screening for its efficient control. Acta Phytophy Sin. 47, 1333–1342. 10.13802/j.cnki.zwbhxb.2020.2019211

[B61] Xu L. H. Ravnskov S. Larsen J. Nilsson R. H. Nicolaisen M. (2012). Soil fungal community structure along a soil health gradient in pea fields examined using deep amplicon sequencing. Soil Biol. Biochem. 46, 26–32. 10.1016/j.soilbio.2011.11.010

[B62] Xu S. Liu Y. X. Cernava T. Wang H. K. Zhou Y. Q. Xia T. (2022). Fusarium fruiting body microbiome member *Pantoea agglomerans* inhibits fungal pathogenesis by targeting lipid rafts. Nat. Microbiol. 7, 831–843. 10.1038/s41564-022-01131-x 35618775

[B63] Zhang J. Y. Liu Y. X. Zhang N. Hu B. Jin T. Xu H. R. (2019). *NRT1.1B* is associated with root microbiota composition and nitrogen use in field-grown rice. Nat. Biotechnol. 37, 676–684. 10.1038/s41587-019-0104-4 31036930

[B64] Zheng H. P. Yang T. J. Bao Y. Z. He P. P. Yang K. M. Mei X. L. (2021). Network analysis and subsequent culturing reveal keystone taxa involved in microbial litter decomposition dynamics. Soil Biol. Biochem. 157, 108230. 10.1016/j.soilbio.2021.108230

[B65] Zhou X. G. Zhang X. H. Ma C. L. Wu F. Z. Jin X. Dini-Andreote F. (2022). Biochar amendment reduces cadmium uptake by stimulating cadmium-resistant PGPR in tomato rhizosphere. Chemosphere 307, 136138. 10.1016/j.chemosphere.2022.136138 36002065

