# Supplementary material for: Microbiome analysis and biocontrol bacteria isolation from rhizosphere soils associated with different sugarcane root rot severity
Source: Front Bioeng Biotechnol. 2022 Dec 16;10:1062351. doi: 10.3389/fbioe.2022.1062351 (PMC9802638; doi:10.3389/fbioe.2022.1062351)
Supplement: Supplementary file 1 [file DataSheet1.PDF]

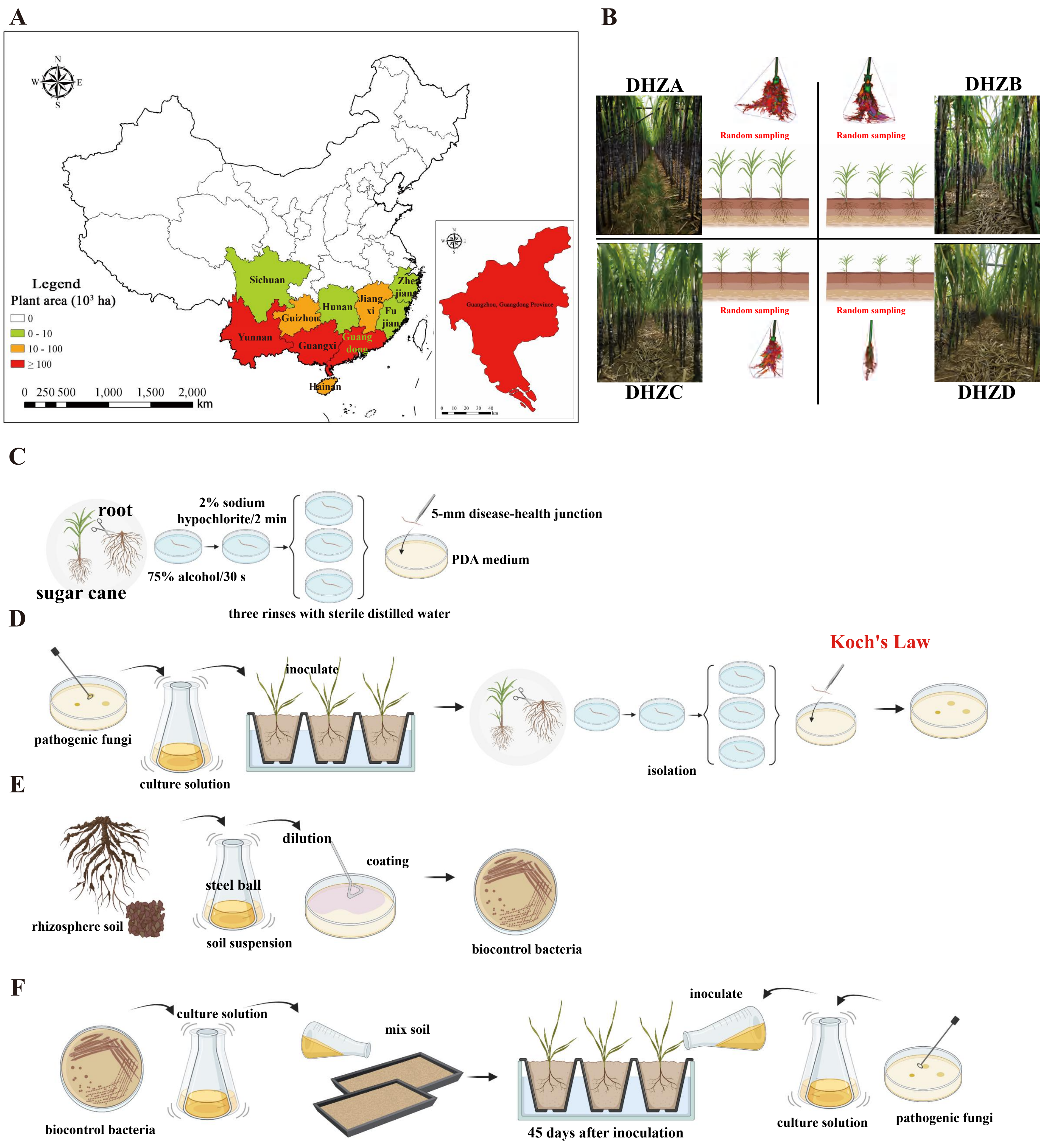

Figure S1

Experimental design schemes. (A) Sugarcane planting area in China, and the sampling location of sugarcane root rot (inset). (B) Categorization of sugarcane root rot severity based on visual assessment of plant height and stoutness (representative photographs is displayed). Sampling of roots and rhizosphere soils was random, as illustrated by the schemes. Healthy (DHZA), mildly diseased (DHZB), moderately diseased (DHZC) and severely diseased (DHZD). "DHZ" is short for "Dahuizhong", the cultivar name called by the local farmer. (C) Experimental procedure for fungal pathogen isolation. (D) Validation of isolated fungal strains as the pathogen causing sugarcane root rot, according to Koch's law. (E) Screening of rhizosphere soil bacteria by soil dilution isolation method. (F) A pot experiment for assessing biocontrol effect of the isolated bacterial strains.

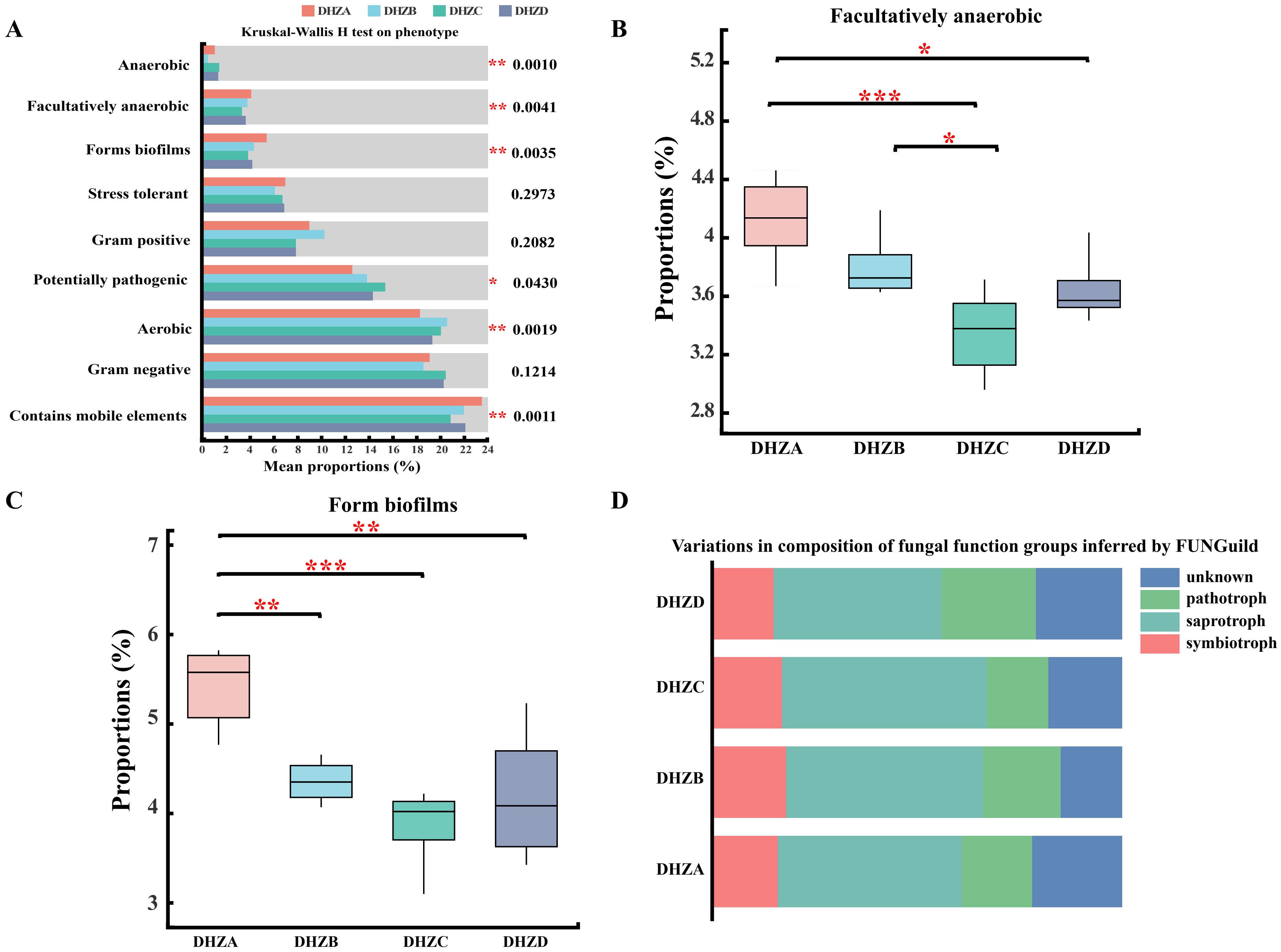

Figure S2

Prediction of the rhizosphere soil microbiome. (A) The Kruskal-Wallis H test of the BugBase predicted phenotypes of the bacterial community in different rhizosphere soils. *$P < 0.05$, **$P < 0.01$, ***$P < 0.001$. (B and C) Phenotypic differences of facultatively anaerobic and forming biofilms in the bacterial community from different rhizosphere soil samples. *$P < 0.05$, **$P < 0.01$, ***$P < 0.001$. (D) FUNGuild prediction of rhizosphere soil fungal community of different samples.

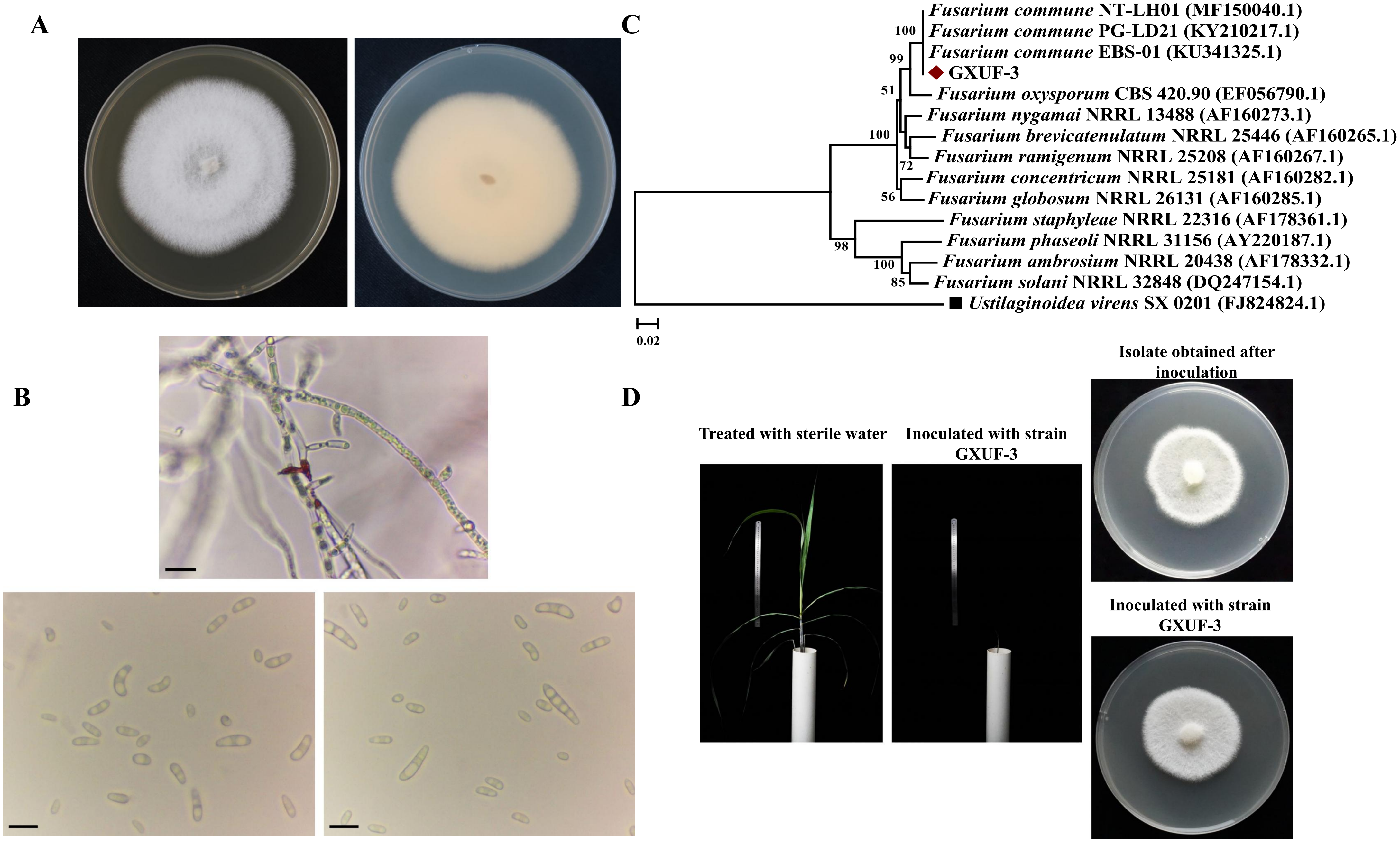

Figure S3

Identification of *Fusarium* pathogen causing sugarcane root rot. (A) Front and back morphology of GXUF-3 colony, grown on PDA medium for 5 d. (B) Microscopic observation of GXUF-3 mycelia and spores. Scale bar = 50 µm. (C) Phylogenetic analysis of GXUF-3 strain and *Fusarium* species, using the neighborhood-joining method. (D) Koch'law validation of GXUF-3 strain as the causal pathogen of sugarcane root rot.

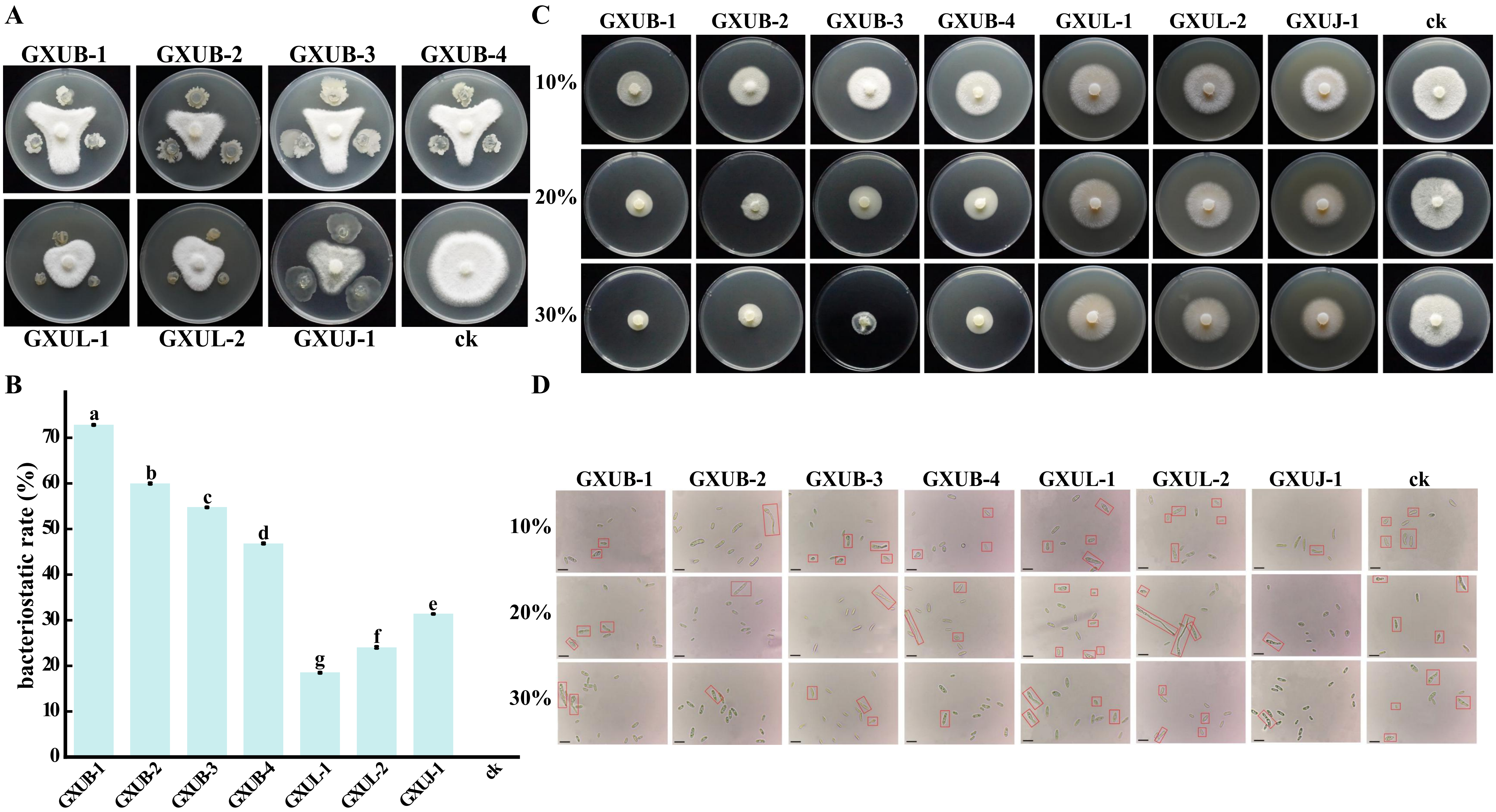

Figure S4

Screening of biocontrol bacteria against GXUF-3 strain. (A) The confrontation culture of different biocontrol bacteria with GXUF-3 strain. (B) Quantification of antibacterial effect of different biocontrol strains on GXUF-3 strain, based on confrontation assay in (A). Different lowercase letters denote significant differences at the $P < 0.05$ level by Duncan's new multiple range test. (C) GXUF-3 strain was allowed to grow on PDA medium containing different concentration of biocontrol bacterial fermentation broth. (D) GXUF-3 spores were allowed to germinate under different concentration of biocontrol bacterial fermentation broth. Red rectangles denote germinated spores, scale bar = 50 μm.
